# Holmium laser versus cold knife visual internal urethrotomy for management of short segment urethral stricture: a prospective randomized clinical trial

**DOI:** 10.1007/s00345-023-04434-8

**Published:** 2023-05-31

**Authors:** Maged M. Ali, Mostafa Kamel, Ahmed Ragab, Abdulbasit Abd Alraheem, Ahmed Sakr

**Affiliations:** Urology Department, Faculty of Medicine, Zagazig University, Zagazig, Egypt

**Keywords:** Urethral stricture, Holmium laser, Urethrotomy, And recurrence

## Abstract

**Objectives:**

To report the safety and efficacy of holmium laser and compare its results with cold knife visual internal urethrotomy (VIU) in the management of short segment urethral stricture.

**Methods:**

This prospective randomized study included 66 male patients aged more than 18 years, with short segment bulbar urethral strictures < 2 cm from March 2020 to March 2022. The patients were randomized into two groups each containing 33 patients. In group A (Cold knife group), Sachse cold knife was used for stricture treatment. In group B (Holmium group), internal urethrotomy was done with Ho:YAG laser. Patients were evaluated before the operation and followed up after the operation at 1, 3, 6 and 12 months by physical examination, IPSS, PVR, Qmax and retrograde urethrography.

**Results:**

There was significant improvement in the mean values of IPSS, PVR and Qmax in both groups. There was no significant difference between both groups in the mean values of IPSS, PVR and Qmax during follow-up visits. However, at the end of follow-up at one year there was statistically significant difference between both groups in the mean values of IPSS, PVR and Qmax due to higher recurrence rate in cold knife group than laser group. The overall complication rate is significantly lower in laser group (*p* = 0.014).

**Conclusion:**

Holmium laser VIU is an effective and safe treatment option for short segment urethral stricture with shorter operative time, less complication rate and less recurrence than cold knife VIU.

## Introduction

Urethral stricture has been diagnosed in humans since ages being recorded in the ancient literature of Hindus, Pharaohs and Greeks. Its treatment is very difficult to be satisfactory for the patient [1, 2]. Different treatment modalities have been tried for management of urethral strictures ranging from simple noninvasive techniques to one-stage or more urethroplasty depending on its length, location, depth of scar and extension of spongio-fibrosis [3, 4]. It includes dilatation, blind or direct vision urethrotomy, stent placement, urethroplasty with or without flaps or grafts and salvage perineal urethrostomy [5–8]. Urethrotomy was first described by Otis and Mauermayer in the nineteenth century [9]. The first performed direct visual urethrotomy was in 1957 by Ravasini who described internal urethrotomy with incision of the stricture using electrocautery with significant inevitable thermal effect on healthy surrounding tissues [10]. Sachse from Germany in 1971 introduced the urethrotome with its sharp bladed cold knife reporting 80% success rate [11]. Although urethroplasty remained the gold standard for treatment of urethral stricture disease, visual internal urethrotomy (VIU) gained its popularity among urologists being easy, simple, rapid and with short convalescence with limited indications [12–14]. Lasers started to be used in the management of urethral strictures aiming for improving results. Different kinds of laser energy have been used including carbon dioxide, argon, diode, excimer, Nd:YAG (neodymium-doped yttrium aluminum garnet), KTP (potassium titanyl phosphate) and Ho:YAG (holmium-doped yttrium aluminum garnet). For a period of time, none of these types has been shown to be better than the others [15]. Ho:YAG is the newly introduced member in the spectrum of laser types in urethrotomy; it gives both vaporization and cutting by direct contact with minimal penetration and forward scattering [16]. This prospective study aimed to evaluate the efficacy and safety of Ho:YAG laser versus cold knife in the management of short segment bulbar urethral stricture.

## Patients and methods

This prospective study was conducted in the Urology department at our institute from March 2020 to March 2022 on 80 male patients with bulbar urethral strictures < 2 cm. After Institutional Review Board (IRB) approval and written informed consent was obtained from all patients. After that, patients were randomly allocated by a closed envelop method in two groups: group A (cold knife urethrotomy group) and group B (Ho:YAG laser urethrotomy group). Pediatric age group, patients with previous urethral surgery or urethral dilatation, patients with multiple strictures, patients with skeletal deformity hindering lithotomy position, patient who were unfit for surgery and/or anesthesia, patients with bleeding tendency and/or coagulopathy were excluded from the study. All patients were assessed by a thorough history taking with International Prostate Symptom Score (IPSS), full physical examination, renal and liver function tests, complete blood picture, coagulation profile, urine culture and sensitivity to ensure sterile urine before the procedure, preoperative uroflowmetry results including the maximum flow rate (Qmax) and mean flow rate (Qmean) values, and retrograde urethrography (RGU) with voiding films. Both pelvi-abdominal ultrasound by 3 MHz transducer for estimation of post-voiding residual (PVR) urine and sono-urethrogram by 7.5 MHz transducer for detection of degree of spongio-fibrosis were done.

### Operative technique

The procedure was carried out by one surgeon under spinal anesthesia in lithotomy position with padding of pressure areas. Perioperative antibiotics were given to all patients. Initial urethrocystoscopy was done using (16 fr) diagnostic cystoscope (Karl Storz, Germany) under video monitoring with placement of (5 fr) ureteral catheter to measure the stricture length and a (0.035 mm) guide wire fixed into the urinary bladder. In group A, the urethrotome was advanced in the urethra through (22 fr) cystoscopy sheath up to the stricture site. The incision was done using the cold knife at 12 o’clock position. The procedure was repeated till the stricture appeared to be opened up. Once the stricture was ablated, the diagnostic cystoscopy was passed into the urinary bladder. Bladder was emptied and safety wire may be removed. In group B, the machine used was Ho:YAG laser device (Sphinx 100 W, holmium-YAG laser, LISA Laser Products–OHG, Germany) with setting of 15 W power (2 J and 15 Hz frequency). The holmium laser fiber 550 µm was introduced through the (22 fr) cystoscopy sheath. Incision was done with the laser fiber at 12 o’clock. Once the stricture was ablated, the diagnostic cystoscopy was passed into the urinary bladder. Bladder was emptied and safety wire may be removed. In all patients of both groups, Foley’s catheter (16 fr) was inserted per urethra for approximately 5 days. Operative time was calculated from the beginning of insertion of cystoscopy sheath from external urethral meatus involving the treatment of stricture site to the removal of working endoscopy from urethral meatus and catheter fixation.

### Postoperative assessment

All patients of both groups were evaluated for intra- or postoperative complications, e.g., bleeding, fever or postoperative pain by visual analog scale (VAS). All patients were discharged on the second day of the operation.

### Follow-up and outcome measurements

Patients were followed up after catheter removal and at 1, 3, 6 and 12 months after the operation. All patients were evaluated in each follow-up visit with IPSS, ultrasound and uroflowmetry. Retrograde urethrogram was done at 6 and 12 months of follow-up period. Successful treatment means spontaneous voiding without any persistent symptoms or significant PVR with Qmax > 15 mL/s without any requirement of auxiliary manoeuver. Failure was defined as the presence of obstructive lower urinary tract symptoms, Qmax < 10 mL/s, recurrent stricture by retrograde urethrogram or the need for any auxiliary procedure including dilatation, another internal urethrotomy or urethroplasty.

### Statistical analysis

Data were analyzed by Statistical Package for the Social Sciences (SPSS version 20.0) software for analysis. Data were tested for normal distribution using the Shapiro–Walk test. According to the type of data, categorical data were represented by number and percentage, while quantitative data were represented by mean ± SD. Differences among quantitative independent groups were tested by independent *t* test when normally distributed and Mann–Whitney *U* test when the data were not normally distributed. While differences among qualitative independent multiple groups were tested by Chi-square test. Repeated-measures ANOVA test was used for comparison between preoperative and different follow-up visits postoperative results. Post hoc analysis using the Bonferroni test was done when there were significant differences in the serial measurements in each group. *p* value was set at < 0.05 for significant results and < 0.001 for high significant results.

## Results

**Fig. 1:**
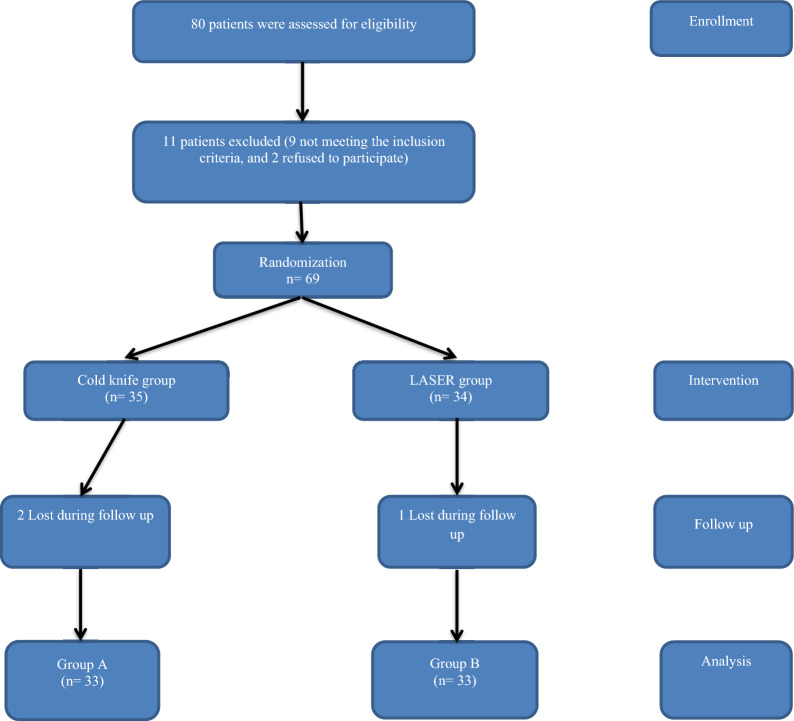
Flow chart of the patients

Sixty-six patients completed this prospective randomized study and follow-up (33 patients in each group). The patients flowchart in the study is shown in Fig. 1. The mean age of patients in both groups was (44.23 ± 12.04 and 42.58 ± 9.32 years), respectively. No significant difference was present between both groups regarding patients and strictures characteristics including its length or degree of spongio-fibrosis (Table 1). In cold knife group, there was highly significant improvement in IPSS, PVR and Qmax at 1, 3, 6 and 12 months from the preoperative measurements (*p* < 0.001). Also, in laser group, there was highly significant improvement in IPSS, PVR and Qmax at 1, 3, 6 and 12 months from the preoperative measurements (*p* < 0.001). There was no significant difference between both groups in follow-up mean values of IPSS, PVR and Qmax at 1, 3 and 6 months; however at the end of follow-up at 12 months, there was significant difference (*p* = 0.028, *p* = 0.021, and *p* = 0.047, respectively) as shown in Tables 2 and 3. Operative time was significantly shorter (*p* < 0.001) in laser group (Table 2). The overall complication rate was significantly lower (*p* = 0.014) in laser group (Table 2). At the end of follow-up period, the recurrence rate was significantly lower (*p* = 0.021) in laser group (Table 2). Post hoc analysis of preoperative and postoperative mean values of IPSS, Qmax and PVR in group A (Cold Knife group) showed that the preoperative measurements were the worst, while no significant difference between 1, 3, 6 and 12 months measurements. Also, post hoc analysis of preoperative and postoperative mean values of IPSS, PVR and Qmax in group B (LASER group) showed that the preoperative measurements were the worst while no significant difference between 1, 3, 6 and 12 months measurements.

**Table 1:** Patient demographics and clinical data

	Group A Cold Knife group	Group B LASER group	*p* value
Continuous data, mean ± SD			
Age (years)	44.23 ± 12.04	42.58 ± 9.32	0.659*
BMI (kg/m^2^)	29.94 ± 3.83	29.17 ± 3.72	0.561*
ASA score	1.2 ± 0.5	1.3 ± 0.4	0.423**
Pre-operative IPSS	24.88 ± 2.54	25.05 ± 2.48	0.839*
Pre-operative PVR (mL)	257.94 ± 58.17	260.0 ± 57.11	0.918*
Pre-operative Qmax (mL/s)	6.58 ± 1.69	6.88 ± 1.76	0.624*
Stricture length (cm)	1.16 ± 0.29	1.15 ± 0.33	0.708*
Categorical data, *N* (%)			
Degree of spongio-fibrosis			0.158†
Mild	22 (66.7)	20 (60.6)	
Moderate	10 (30.3)	13 (39.4)	
Severe	1 (3)	1 (3)	
Possible etiology			0.708†
Traumatic	5 (15.1)	6 (18.2)	
Inflammatory	2 (6.1)	2 (6.1)	
Post-catheterization	24 (72.7)	22 (66.6)	
Idiopathic	2 (6.1)	3 (9.1)	

*BMI* body mass index, *ASA* American Society of Anaesthesiologists, *IPSS* International prostate symptom score, *PVR* post-void residual urine, *Qmax* maximum flow in uroflowmetry

*Independent *t* test

**Mann–Whitney *U* test

^†^Chi-square test

**Table 2:** Operative data and clinical outcomes

	Group A Cold Knife group	Group B LASER group	*p* value
Continuous data, mean ± SD			
Operative time (min)	26.29 ± 4.34	18.11 ± 3.92	< 0.001*
Post-operative IPSS			
1 month	4.17 ± 1.39	4.47 ± 1.17	0.849**
3 months	4.15 ± 1.95	4.24 ± 2.21	0.544**
6 months	4.22 ± 1.85	4.25 ± 2.27	0.874**
12 months	6.82 ± 1.57	4.81 ± 1.74	0.028**
Post-operative PVR (mL)			
1 month	41.85 ± 13.87	39.35 ± 12.6	0.682*
3 months	41.72 ± 13.22	38.87 ± 11.58	0.099*
6 months	45.22 ± 13.85	42.25 ± 12.27	0.374*
12 months	63.28 ± 17.58	43.88 ± 15.25	0.021*
Post-operative Qmax (mL/s)			
1 month	18.71 ± 2.17	18.88 ± 2.2	0.692*
3 months	18.88 ± 2.84	18.91 ± 3.06	0.816*
6 months	17.21 ± 2.85	17.95 ± 3.07	0.811*
12 months	14.37 ± 3.08	16.12 ± 3.11	0.047*
Hospital stay (h)	9.53 ± 0.8	9.14 ± 1.1	0.489*
Postoperative pain (VAS score)	3.2 ± 0.8	3.1 ± 0.9	0.436*
Categorical data, *N* (%)			
Complications			0.014^†^
Bleeding per urethra	4 (12.1)	2 (6.1)	
Fever	2 (6.1)	1 (3)	
Extravasation	3 (9.1)	1 (3)	
UTI	4 (12.1)	2 (6.1)	
1 year recurrence rate	6 (18.2)	3 (9.1)	0.021^†^

*IPSS* International prostate symptom score, *PVR* post-void residual urine, *Qmax* maximum flow in uroflowmetry, *VAS score* visual Analog Scale, *UTI* urinary tract infection

*Independent *t* test

**Mann Whitney *U* test

^†^Chi-square test

**Table 3:** Post HOC analysis: comparison between serial measurement of IPSS, PVR and Qmax in each group

	Group A (Cold Knife group)			Group B (LASER group)		
	Time	Time	*p* value	Time	Time	*p* value
IPSS	Pre-operative	1 month after	< 0.001	Pre-operative	1 month after	< 0.001
		3 months after	< 0.001		3 months after	< 0.001
		6 months after	< 0.001		6 months after	< 0.001
		12 months after	< 0.001		12 months after	< 0.001
	1 month after	Pre-operative	< 0.001	1 month after	Pre-operative	< 0.001
		3 months after	0.732		3 months after	0.793
		6 months after	0.664		6 months after	0.779
		12 months after	0.511		12 months after	0.559
	3 months after	Pre-operative	< 0.001	3 months after	Pre-operative	< 0.001
		1 month after	0.732		1 month after	0.793
		6 months after	0.881		6 months after	0.488
		12 months after	0.752		12 months after	0.686
	6 months after	Pre-operative	< 0.001	6 months after	Pre-operative	< 0.001
		1 month after	0.664		1 month after	0.779
		3 months after	0.881		3 months after	0.488
		12 months after	0.941		12 months after	0.866
PVR	Pre-operative	1 month after	< 0.001	Pre-operative	1 month after	< 0.001
		3 months after	< 0.001		3 months after	< 0.001
		6 months after	< 0.001		6 months after	< 0.001
		12 months after	< 0.001		12 months after	< 0.001
	1 month after	Pre-operative	< 0.001	1 month after	Pre-operative	< 0.001
		3 months after	0.234		3 months after	0.311
		6 months after	0.776		6 months after	0.488
		12 months after	0.324		12 months after	0.424
	3 months after	Pre-operative	< 0.001	3 months after	Pre-operative	< 0.001
		1 month after	0.234		1 month after	0.311
		6 months after	0.456		6 months after	0.566
		12 months after	0.567		12 months after	0.559
	6 months after	Pre-operative	< 0.001	6 months after	Pre-operative	< 0.001
		1 month after	0.776		1 month after	0.488
		3 months after	0.456		3 months after	0.566
		12 months after	0.911		12 months after	0.822
Qmax	Pre-operative	1 month after	< 0.001	Pre-operative	1 month after	< 0.001
		3 months after	< 0.001		3 months after	< 0.001
		6 months after	< 0.001		6 months after	< 0.001
		12 months after	< 0.001		12 months after	< 0.001
	1 month after	Pre-operative	< 0.001	1 month after	Pre-operative	< 0.001
		3 months after	0.449		3 months after	0.178
		6 months after	0.499		6 months after	0.298
		12 months after	0.454		12 months after	0.871
	3 months after	Pre-operative	< 0.001	3 months after	Pre-operative	< 0.001
		1 month after	0.449		1 month after	0.178
		6 months after	0.938		6 months after	0.936
		12 months after	0.788		12 months after	0.444
	6 months after	Pre-operative	< 0.001	6 months after	Pre-operative	< 0.001
		1 month after	0.499		1 month after	0.298
		3 months after	0.938		3 months after	0.936
		12 months after	0.602		12 months after	0.668

*IPSS* International prostate symptom score, *PVR* post-void residual urine, *Qmax* maximum flow in uroflowmetry

## Discussion

Laser invaded medicine in the last decades of the past century. Soon after that different laser technologies were widely used for urology purposes. It gained popularity among urologists in endoscopic resection of benign prostatic hyperplasia or bladder tumors, fragmenting renal or ureteric stones, and lastly in treatment of urethral strictures because of less bleeding, shorter hospitalization time and less complication rate [17]. Urethral stricture is a disease with a potentially high recurrence risk that forced urologists to use many substances for intra-lesional injection with conventional VIU like mitomycin-c, methylprednisolone and the tetra-inject (triamcinolone, hyaluronidase, mitomycin-c and N-acetyl cysteine) [18, 19]. Two main principles should be taken in mind when treating urethral stricture to minimize the risk of recurrence: removal of fibrotic tissues and avoidance of injury of healthy tissues [20]. Holmium laser with its high wavelength of 2140 nm and short emission time of 0.25 mL/s provides a good option for urethral strictures management with vaporization of fibrotic scarred tissues and minimal thermal damage for normal tissues [21]. The largest meta-analysis presented by Jin et al. in 2010 found that laser urethrotomy results were better but without statistically significant difference between laser urethrotomy and with the conventional cold knife VIU [22]. With increased popularity of laser urethrotomy use, the number of publications studying and comparing the two maneuvers has recently increased with high emphasis on that laser urethrotomy is more effective and more safe [23].

In the current study, we found that laser group had shorter operative time than cold knife group with significant difference between studied groups (18.11 ± 3.92 min and 26.29 ± 4.34 min), respectively, which was in agreement with many studies [23–26]. However, Yenice et al. in their study found that the operative time for laser group was (21.9 ± 3.8 min) which is longer than cold knife group (18.4 ± 2.3 min) [27]. The difference between these results may be related to technical difficulty and lack of experience for laser treatment.

In our study, we found that there was dramatic improvement in the mean values of IPSS, PVR and Qmax in both groups. There was no significant difference between both groups in the mean values of IPSS, PVR and Qmax during follow-up visits. However at the end of follow-up at 1 year, there was statistically significant difference between both groups in the mean values of IPSS, PVR and Qmax due to higher recurrence rate in cold knife group than laser group. These results were matching with results of many studies even using other types of laser [23, 25, 28–30]. The overall complication rate in our study is significantly lower in laser group than in cold knife group (*p* = 0.014) and also this is in agreement with different studies comparing complications of laser and cold knife urethrotomy [31, 32].

In fact, urethroplasty is the best treatment option for urethral stricture removing all the scarred and fibrotic tissues which is the corner stone in preventing recurrence. However, urologists depend on VIU for its ease, simplicity, less invasiveness, short convalescence and suitability in short segment strictures. The main obstacle in the conventional VIU is inability to remove the fibrotic tissues. Endo-urologists luckily found relief in holmium laser that vaporizes an impeachable part of the scarred tissues without affecting healthy tissues as its penetration depth is only 0.4 mm.

### Limitations of this study

The main limitation of this study is that the surgeon knows which arm the patient belongs to. This bias is inevitable, unfortunately. Another limitation was the relatively small sample size and relatively short follow-up period. So, we recommend further studies with larger sample size and longer duration of follow-up.

## Conclusion

Holmium laser VIU is an effective and safe treatment option for short segment urethral stricture with shorter operative time, less complication rate and less recurrence than cold knife VIU.

## Data Availability

Not applicable.
